# Supplementary material for: Discovery and application of insertion-deletion (INDEL) polymorphisms for QTL mapping of early life-history traits in Atlantic salmon
Source: BMC Genomics. 2010 Mar 8;11:156. doi: 10.1186/1471-2164-11-156 (PMC2838853; doi:10.1186/1471-2164-11-156)
Supplement: Additional file 2 — Information on developed 76 locus single-run INDEL panel in Atlantic salmon. Information on fluorescence labeling, primer concentrations, PCR pooling and links to alignments, INDEL motifs and GENESCAN (Burge and Karlin 1997) predictions of genes/exons are available in html format. [file 1471-2164-11-156-S2.ZIP › Additionalfile2/snpsummary4246.html]

```
Cluster 977 Contig 3

prev  Summary    Contig List  next
```

Size of Consensus sequence = 1921

Number of sequences = 27

Minimum redundancy = 6

Key

A gi|54021317|gb|CV429108.1|CV429108 EST02337 Atlantic salmon Lambda Zap Express heart cDNA library Salmo salar cDNA clone hr\_008\_d07 5', mRNA sequence  
B gi|24355218|gb|CA049048.1|CA049048 ssalkspa003051 kidney Salmo salar cDNA, mRNA sequence  
C gi|117487927|gb|EG820144.1|EG820144 EST\_ssal\_evd\_17250 ssalevd thymus Salmo salar cDNA Salmo salar cDNA clone ssal\_evd\_522\_019\_rev 5', mRNA sequence  
D gi|29313537|gb|CB502311.1|CB502311 ssalmge501125 gut Salmo salar cDNA, mRNA sequence  
E gi|24382426|gb|CA052183.1|CA052183 ssalrga514317 mixed\_tissue Salmo salar cDNA, mRNA sequence  
F gi|24383263|gb|CA053020.1|CA053020 ssalrgb515222 mixed\_tissue Salmo salar cDNA, mRNA sequence  
G gi|89844217|gb|DY700373.1|DY700373 EST\_ssal\_rgb2\_56112 ssalrgb2 mixed\_tissue Salmo salar cDNA Salmo salar cDNA clone ssal\_rgb2\_590\_258\_fwd 3', mRNA sequence  
H gi|85047496|gb|DW575674.1|DW575674 EST\_ssal\_rgb2\_40093 rgb2 Salmo salar cDNA clone ssal\_rgb2\_565\_061\_fwd 3', mRNA sequence  
I gi|84986872|gb|DW537222.1|DW537222 EST\_ssal\_rgb2\_1641 rgb2 Salmo salar cDNA clone ssal\_rgb2\_503\_294\_fwd 3', mRNA sequence  
J gi|117852997|gb|EG925693.1|EG925693 EST\_ssal\_evf\_28281 ssalevf mixed\_tissue Salmo salar cDNA Salmo salar cDNA clone ssal\_evf\_537\_190\_fwd 3', mRNA sequence  
K gi|117852996|gb|EG925692.1|EG925692 EST\_ssal\_evf\_28280 ssalevf mixed\_tissue Salmo salar cDNA Salmo salar cDNA clone ssal\_evf\_537\_190\_rev 5', mRNA sequence  
L gi|117450688|gb|EG782907.1|EG782907 EST\_ssal\_evd\_39400 ssalevd thymus Salmo salar cDNA Salmo salar cDNA clone ssal\_evd\_552\_280\_rev 5', mRNA sequence  
M gi|117513908|gb|EG845667.1|EG845667 EST\_ssal\_eve\_58392 ssaleve thyroid Salmo salar cDNA Salmo salar cDNA clone ssal\_eve\_579\_224\_rev 5', mRNA sequence  
N gi|117540993|gb|EG872438.1|EG872438 EST\_ssal\_eve\_33387 ssaleve thyroid Salmo salar cDNA Salmo salar cDNA clone ssal\_eve\_545\_123\_rev 5', mRNA sequence  
O gi|24463294|gb|BQ035913.1|BQ035913 SL2-0113 Atlantic Salmon liver SSH Salmo salar cDNA clone SL2-0113 similar to other reported ESTs, mRNA sequence  
P gi|117513907|gb|EG845666.1|EG845666 EST\_ssal\_eve\_58391 ssaleve thyroid Salmo salar cDNA Salmo salar cDNA clone ssal\_eve\_579\_224\_fwd 3', mRNA sequence  
Q gi|117506222|gb|EG837981.1|EG837981 EST\_ssal\_eve\_12475 ssaleve thyroid Salmo salar cDNA Salmo salar cDNA clone ssal\_eve\_516\_371\_rev 5', mRNA sequence  
R gi|117538580|gb|EG870025.1|EG870025 EST\_ssal\_eve\_31215 ssaleve thyroid Salmo salar cDNA Salmo salar cDNA clone ssal\_eve\_542\_145\_rev 5', mRNA sequence  
S gi|117538581|gb|EG870026.1|EG870026 EST\_ssal\_eve\_31216 ssaleve thyroid Salmo salar cDNA Salmo salar cDNA clone ssal\_eve\_542\_145\_fwd 3', mRNA sequence  
T gi|84975476|gb|DW473877.1|DW473877 SGP315853 Atlantic salmon Spleen cDNA library Salmo salar cDNA clone MI5-4090 5', mRNA sequence  
U gi|117506221|gb|EG837980.1|EG837980 EST\_ssal\_eve\_12474 ssaleve thyroid Salmo salar cDNA Salmo salar cDNA clone ssal\_eve\_516\_371\_fwd 3', mRNA sequence  
V gi|117450689|gb|EG782908.1|EG782908 EST\_ssal\_evd\_39401 ssalevd thymus Salmo salar cDNA Salmo salar cDNA clone ssal\_evd\_552\_280\_fwd 3', mRNA sequence  
W gi|85016922|gb|DW545578.1|DW545578 EST\_ssal\_rgb2\_9997 rgb2 Salmo salar cDNA clone ssal\_rgb2\_517\_033\_rev 5', mRNA sequence  
X gi|84986871|gb|DW537221.1|DW537221 EST\_ssal\_rgb2\_1640 rgb2 Salmo salar cDNA clone ssal\_rgb2\_503\_294\_rev 5', mRNA sequence  
Y gi|117540992|gb|EG872437.1|EG872437 EST\_ssal\_eve\_33386 ssaleve thyroid Salmo salar cDNA Salmo salar cDNA clone ssal\_eve\_545\_123\_fwd 3', mRNA sequence  
Z gi|85047495|gb|DW575673.1|DW575673 EST\_ssal\_rgb2\_40092 rgb2 Salmo salar cDNA clone ssal\_rgb2\_565\_061\_rev 5', mRNA sequence  
a gi|89844215|gb|DY700372.1|DY700372 EST\_ssal\_rgb2\_56111 ssalrgb2 mixed\_tissue Salmo salar cDNA Salmo salar cDNA clone ssal\_rgb2\_590\_258\_rev 5', mRNA sequence

7 SNPs detected

A B C D E F G H I J K L M N O P Q R S T U V W X Y Z a  cosegregation weighted

421 . . T T T A A T T A A T A A . A A - - . A . . . . . .   1/7 8.99
423 . . - - - A - - - A A - A - A A - - - . - . . . . . .   1/7 9.52
489 . . T T T - - T T - - T - - - - - - - . - T . . . . .   3/7 30.16
490 . . A A A - - A A - - A - - - - - - - . - A . . . . .   3/7 30.16
551 . . C C C - - C C - - C - - C - C - - C C C - C . . .   2/7 23.28
552 . . A A A - - A A - - A - - A - A - - A A A - A . . .   2/7 23.28
569 . . C C C T T C C T T C T T T T T T T T T C T C . . .   3/7 34.92
